# Photoprotective Effects of a Hyperoside-Enriched Fraction Prepared from *Houttuynia cordata* Thunb. on Ultraviolet B-Induced Skin Aging in Human Fibroblasts through the MAPK Signaling Pathway

**DOI:** 10.3390/plants10122628

**Published:** 2021-11-29

**Authors:** Sariya Mapoung, Sonthaya Umsumarng, Warathit Semmarath, Punnida Arjsri, Kamonwan Srisawad, Pilaiporn Thippraphan, Supachai Yodkeeree, Pornngarm Dejkriengkraikul

**Affiliations:** 1 Department of Biochemistry, Faculty of Medicine, Chiang Mai University, Chiang Mai 50200, Thailand; srmapoung@gmail.com (S.M.); warathit_semmarath@cmu.ac.th (W.S.); punnida_dream@hotmail.com (P.A.); k.srisawad@gmail.com (K.S.); tipprapant@gmail.com (P.T.); yodkeelee@hotmail.com (S.Y.); 2 Center for Research and Development of Natural Products for Health, Chiang Mai University, Chiang Mai 50200, Thailand; sonthaya.u@cmu.ac.th; 3 Department of Veterinary Biosciences and Veterinary Public Health, Division of Veterinary Preclinical Sciences, Faculty of Veterinary Medicine, Chiang Mai University, Chiang Mai 50200, Thailand

**Keywords:** skin aging, ultraviolet-B, *Houttuynia cordata*, hyperoside-enriched fraction, flavonoid, photoprotective, natural cosmetics

## Abstract

Ultraviolet-B (UVB) irradiation causes skin damage via deleterious effects including oxidative stress, inflammation, and collagen degradation. The photoprotective effects of a hyperoside-enriched fraction obtained from *Houttuynia cordata* Thunb. (*H. cordata*) on the attenuation of UVB-induced skin aging in human fibroblasts were investigated. The solvent-partition technique was used to establish the hyperoside-enriched fraction of *H. cordata* (HcEA). The active compounds identified in the *H. cordata* extracts were hyperoside, quercitrin, chlorogenic acid, and rutin. With regard to the photoprotective effects of *H. cordata* on UVB-irradiated dermal fibroblasts, HcEA and hyperoside inhibited intracellular ROS production and inflammatory cytokine secretions (IL-6 and IL-8), while increasing collagen type I synthesis along with downregulating MMP-1 gene and protein expressions. Mechanistically, the hyperoside-enriched fraction obtained from *H. cordata* inhibited UVB-irradiated skin aging through regulation of the MAPK signaling pathway by attenuating the activation of JNK/ERK/c-Jun in human dermal fibroblasts. The hyperoside-enriched fraction of *H. cordata* exerted potent anti-skin aging properties against UVB exposure. The findings of this study can be applied in the cosmetics industry, as *H. cordata* extract can potentially be used in pharmaceutical or cosmetic formulations as a photoprotective or anti-skin aging agent.

## 1. Introduction

Skin aging is a complex, multifactorial process resulting in functional and esthetic changes in the skin. These changes result from extrinsic processes that include ultraviolet (UV) radiation [1,2]. UV radiation can damage telomeres and induce reactive oxygen species (ROS) production, thereby inducing cellular senescence. These senescent cells secrete a specific set of pro-inflammatory mediators which can cause deep changes in tissue structure and function [3]. Based on UV physiological and biological effects, UV radiation can be further divided into three main bands—the 315–400 nm band (designated as UVA), the 280–315 nm band (designated as UVB), and the 100–280 nm band (designated as UVC) [4]. Although it comprises only a small portion of the total UV radiation, UVB is thought to be more harmful than UVA, since UVB is most active in damaging the skin and can induce photochemical damage in cellular DNA and proteins [5,6]. UVA radiation has its negative effect on the epidermal keratinocytes and dermal fibroblasts and induces long-term changes, while the changes arising from UVB radiation cause visibly dramatic photo-aging effects mainly within the epidermis and it penetrates the upper part of dermis which rather quite concerning in terms of skin aging characteristics [7]. Mechanistically, the direct absorption of UVB photons by DNA can result in the ge neration of pyrimidine dimers, leading to defects in the DNA strand. On the other hands, UVA does not directly alter the structure of DNA as DNA does not strongly absorb radiation in the UVA range. UVA can damage DNA indirectly, via a photosensitized reaction mediated by generating the radical singlet oxygen (^1^O_2_), resulting in purine base modifications [8]. Irradiation of UVB to the skin was found to cause adverse photochemical reactions including sunburn, inflammation, and DNA damage, which then led to gene mutation and skin cancer development [9,10,11]. Moreover, UVB exposure can induce inflammation of the skin as a result of the transcription factor nuclear factor (NF)-κB or activator protein1 (AP–1) activation. This can lead to increases in the proinflammatory cytokines interleukin 1 (IL-1), IL-6 and IL-8 production, which can attract neutrophils to increase oxidative damage by producing free radicals [12,13,14].

Fibroblasts are the most abundant cells of the dermis, and their dysfunction occurring from UVB radiation significantly contributes to skin aging [4]. When reaching the skin surface, UVB initially induces alterations at the epidermal level, where the bulk of UVB is absorbed. It can damage DNA in keratinocytes and melanocytes, and it further induces the production of soluble epidermal factors (cytokines and chemokines) and proteolytic enzymes, which are found in the dermis, where most cells are fibroblasts [6]. Previous studies have reported that exposure to all types of UV radiation can stimulate fibroblasts senescence, wherein these senescent fibroblasts are mainly accumulated in the dermis. Senescent fibroblasts are characterized by an accumulation of double-strand DNA breaks and oxidative DNA damage, as well as a reduced extracellular matrix and an increase in matrix metalloproteinase (MMP) production [6,15,16,17]. Among these dermal proteolytic enzymes, MMP-1 (collagenase-1) plays an important role in the unbalanced turn-over or rapid breakdown of collagen molecules in human inflamed/UV-irradiated skin [18]. Therefore, a reduction of inflammatory responses and the inhibition of abnormal collagen breakdown could possibly prevent skin aging by reducing inflammatory conditions and maintaining collagen homeostasis in UVB-exposed skin.

Nowadays, scientific evidence suggests that phytochemicals can slow down or even prevent aging-associated deterioration of skin appearance and function by targeting cellular pathways crucial for regulating cellular senescence and reducing those inflammatory mediators [7,8,12,13]. *Houttuynia cordata* Thunb. is a native perennial herbaceous plant from the Saururaceae family and has been used as traditional medicine in Asian countries such as China, Japan, South Korea, and Thailand [12,19]. *H. cordata* contains many active compounds including volatile oils, water-soluble polysaccharides, and flavonoids. It has been used as a medicinal plant possessing many biological properties including antioxidant and anti-inflammatory activities [19,20]. Previous studies have reported that flavonoids from the leaf extracts of *H. cordata* exhibited strong antioxidant activities [13]. The ethanolic extract of the aerial parts of *H. cordata* inhibited the production of inflammatory biomarkers IL-6 and NO in A549 lung epithelial cells and MH-S alveolar macrophages [21,22]. Moreover, the ethyl acetate fraction of *H. cordata* revealed intracellular ROS scavenging activity in a concentration-dependent manner in UVB-irradiated HaCaT keratinocytes [23]. Accordingly, *H. cordata* could be applied in the field of cosmetics to prevent skin aging caused by UVB-exposure.

Therefore, this study aimed to investigate the photoprotective skin aging properties of the *H. cordata* extracts and their bioactive polyphenols, including phenolic and flavonoid compounds, carried out in a UVB-irradiated human dermal fibroblasts model by determining the antioxidant activity, anti-inflammation abilities, collagen synthesis and MMP-1 inhibition, as well as to elucidate the representative inhibitory pathway.

## 2. Results

### 2.1. Total Phenolic and Total Flavonoid Contents and Antioxidant Activities of H. cordata Extracts

The ethanolic extraction yielded 79 g (15.8 %yield) of *H. cordata* extract (HcEE). To concentrate the phytochemical contents in *H. cordata*, the extract was further processed using the solvent-partitioned extraction technique, and the *H. cordata* ethyl acetate fraction (HcEA), *H. cordata* dichloromethane fraction (HcDM), and *H. cordata* residual fraction (Hc-H_2_O) were finally obtained. All *H. cordata* extracts phytochemical contents were determined as shown in Table 1. The results indicate that HcEA contained the highest total phenolic content (591.01 ± 7.11 mg of gallic acid equivalents/g extract), followed by HcEE, Hc-H_2_O, and HcDM, respectively. Additionally, HcEA also contained the highest flavonoid contents (433.86 ± 10.16 mg of catechin equivalents /g extract), followed by HcEE, HcDM, and Hc-H_2_O, respectively.

The phytochemical (phenolic and flavonoid) screening method of *H. cordata* extracts assisted in the choice of a candidate fraction for further bioactivity experiments. Additionally, the phytochemical contents in the plant extracts could be responsible for their antioxidant properties. Thus, we further investigated the antioxidant activities of the *H. cordata* extracts. As shown in Table 1, the antioxidant activity of *H. cordata* extracts was assessed using two distinct chemical-based antioxidant methods (ABTS and DPPH assays). As expected, the DPPH and ABTS assays showed that HcEA exhibited the strongest antioxidant activity with an inhibitory concentration at 50% (IC_50_) of 17.64 ± 2.25 µg/mL for the DPPH assay and IC_50_ of 4.96±0.24 µg/mL for the ABTS assay. The IC_50_ values of HcEA for both assays were also significantly stronger than for the other *H. cordata* extracts (*p* < 0.01). All the results above indicated that the solvent-partitioned fraction, HcEA, provided a higher amount of polyphenol contents, as well as greater antioxidant activities.

### 2.2. Active Compound Determination of H. cordata Extracts Using HPLC

**Figure 1:**
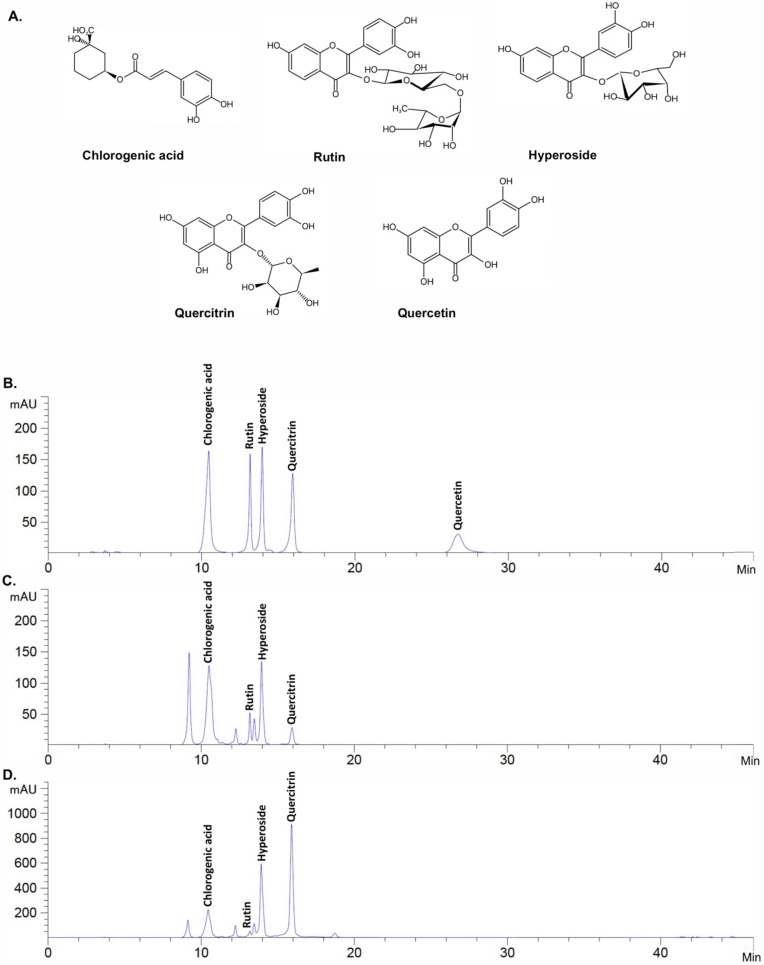
HPLC profile of *H. cordata* extracts. Chemical structure of active compounds determined in this study (chlorogenic acid; rutin; hyperoside; quercitrin; and quercetin, respectively) (**A**). HPLC chromatogram of active compound standards according to their retention time at a concentration of 50 μg/mL for each standard (**B**). HPLC chromatogram of HcEE at 2500 μg/mL (**C**). HPLC chromatogram of HcEA at 2500 μg/mL (**D**). The HPLC chromatograms were evaluated using a reversed-phase C18 column. The mobile phase was composed of mobile phase A (0.1% CH_3_COOH) and mobile phase B (MeCN) under a gradient condition. The detection wavelength was 330 nm. The flow rate was set to 0.8 mL/min.

Previous reports have shown that *H. cordata* contains a wide range of polyphenols, as determined by HPLC [20,24,25]. In brief, rutin, quercetin, hyperoside, quercitrin, and chlorogenic acid were determined using reverse-phased HPLC in polyphenol-rich extract from *H. cordata* [26]. Four flavonoid glycosides (rutin, quercetin, hyperoside, and quercitrin) were identified using HPLC as the major bioactive constituents of the ethanolic extract prepared from the aerial part of *H. cordata* [27]. Therefore, in this study, the main polyphenol compounds (chrologenic acid, rutin, hyperoside, quercitrin, and quercetin) in each fraction were identified by HPLC-DAD. Figure 1 shows the HPLC chromatogram of the standard mixture (Figure 1A), HcEE (Figure 1B), and HcEA (Figure 1C). As three major compounds (hyperoside, quercitrin and chlorogenic acid) were identified from the HPLC chromatogram of HcEE, HcEA, these compounds, together with HcEE and HcEA was selected to determine the cytotoxicity and inhibition of intracellular ROS in UVB-irradiated human dermal fibroblasts in the subsequent experiments. 

### 2.3. H. cordata Thunb. Extracts Displayed No Cytotoxicity on Human Dermal Fibroblasts

**Figure 2:**
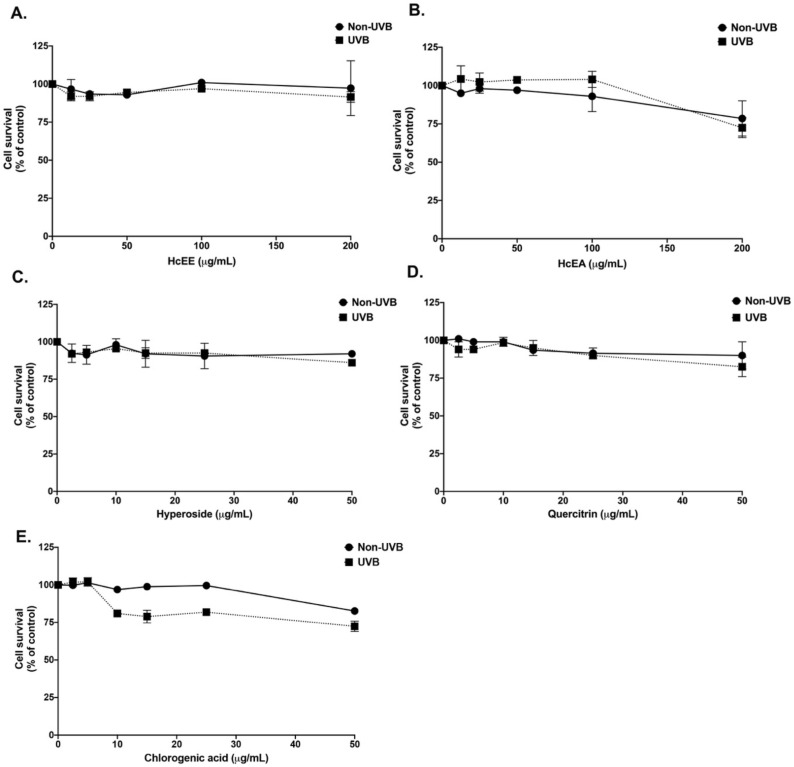
Effects of *H. cordata* extracts and main active compounds on human dermal fibroblast cell viability. Cells were exposed with or without UVB at the intensity of 15 mJ/cm^2^ then treated with HcEE (**A**), HcEA (**B**), hyperoside (**C**), quercitrin (**D**) and chlorogenic acid (**E**) for 48 h. Cell survival was determined using SRB assay. Data are presented as mean ± S.D. values of three independent experiments. HcEE: *H. cordata* ethanolic extract, HcEA: *H. cordata* ethyl acetate fraction, UVB: cells exposed with 15 mJ/cm^2^ of UVB, Non-UVB: control group of cells non-exposed to UVB.

UVB exposure to the skin induces oxidative stress which can cause of inflammatory cascade, resulting in skin aging [28,29]. The cytotoxicity of the HcEE, HcEA, and three major compounds (hyperoside, quercitrin and chlorogenic acid) were investigated by sulforhodamine B (SRB) assay as shown in Figure 2. After 48 h of incubation, HcEE showed no cytotoxicity on human dermal fibroblasts either with or without UVB-irradiation. As shown in Figure 2A,B, there was no noticeable or statistical difference in the fibroblast cytotoxicity with the concentrations lower than 200 μg/mL of HcEE and HcEA (*p* > 0.05). Similarly, hyperoside and quercitrin at concentrations lower than 50 μg/mL showed no cytotoxicity on human fibroblasts, as shown in Figure 2C,D. While chlorogenic acid exhibited slight degree of cytotoxicity on human fibroblasts at a concentration of 50 μg/mL (Figure 2E), a statistically significant difference was not observed (*p* > 0.05). Therefore, the non-toxic concentrations of *H. cordata* extracts (0–100 μg/mL) and the main active compounds (0–20 μg/mL) were used in further studies.

### 2.4. H. cordata Extracts Inhibited Intracellular ROS in UVB-Irradiated Fibroblasts

**Figure 3:**
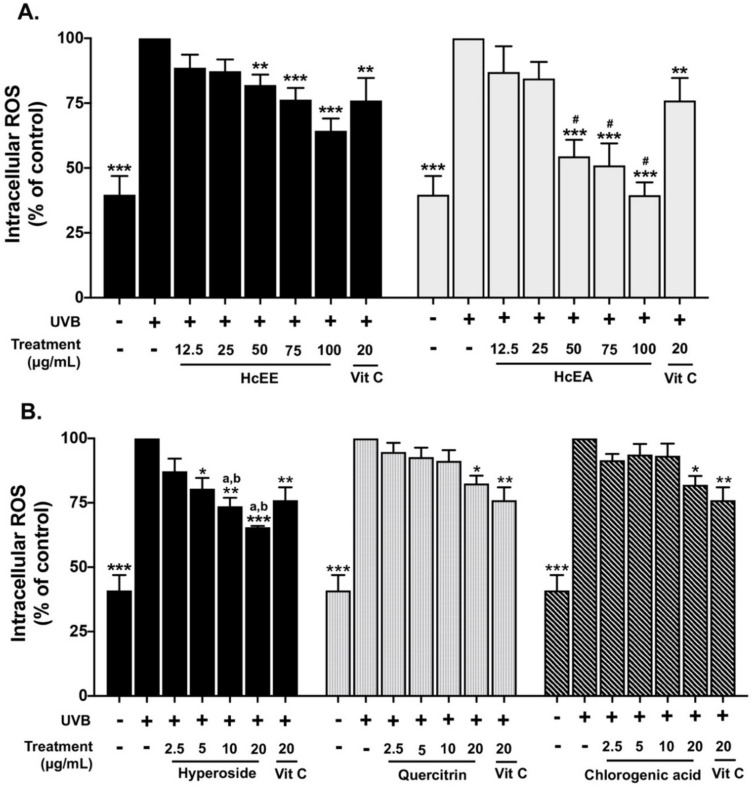
Effects of HcEE, HcEA and active compounds on the inhibition of intracellular ROS in UVB-irradiated human dermal fibroblasts. Cells were exposed with or without UVB 15 mJ/cm^2^, then treated with HcEE, HcEA (**A**), hyperoside, quercitrin and chlorogenic acid (**B**) for 24 h. Intracellular ROS after UVB irradiation in fibroblasts was determined by DCF-DA assay. Vitamin C at 20 μg/mL was used as a positive control. Data are presented as mean ± S.D. values of three independent experiments, * *p* < 0.05, ** *p* < 0.01 and *** *p* < 0.001 vs. the control UVB-irradiated fibroblasts. # *p* < 0.05 vs. HcEE at the same concentration, ^a^ *p* < 0.05 vs. quercitrin at the same concentration, ^b^ *p* < 0.05 vs. chlorogenic acid at the same concentration. HcEE: *H. cordata* ethanolic extract, HcEA: *H. cordata* ethyl acetate fraction, Vit C: vitamin C.

The inhibition of the intracellular ROS production of HcEE, HcEA, and three main active compounds in UVB-irradiated human dermal fibroblasts was examined using a DCF-DA assay as shown in Figure 3. UVB-irradiation at 15 mJ/cm^2^ increased intracellular ROS in human dermal fibroblasts by 2.5-fold when compared with the non-UVB irradiated group. The OD of UVB-irradiated fibroblast control groups was set at 100%. Vitamin C at 20 μg/mL, as a positive control, decreased intracellular ROS by 24% compared to the UVB-irradiated fibroblasts. The HcEE and HcEA treatments inhibited UVB-induced intracellular ROS production in fibroblasts in a dose-dependent manner as shown in Figure 3A. At concentrations of 100 μg/mL, HcEE and HcEA significantly decreased intracellular ROS by 35% (*p* < 0.001) and 61% (*p* < 0.001) when compared with the UVB-irradiated fibroblast control group. Likewise, the HcEA fraction exhibited superior intracellular ROS attenuation in UVB-irradiated fibroblasts when compared with that of the HcEE treatment (*p* < 0.05). Interestingly, the hyperoside treatment of UVB-irradiated fibroblasts exhibited a higher ability to inhibit intracellular ROS production than the quercitrin and chlorogenic acid treatments (*p* < 0.05), as a concentration of 20 μg/mL of hyperoside, quercitrin and chlorogenic acid inhibited intracellular ROS in UVB-irradiated fibroblasts by 41%, 26% and 21%, respectively, when compared to the UVB-irradiated control group (Figure 3B).

### 2.5. Inhibitory Effects of HcEE, HcEA, and Its Bioactive Compounds on the Pro-Inflammatory Cytokine in UVB-Irradiated Human Dermal Fibroblasts

**Figure 4:**
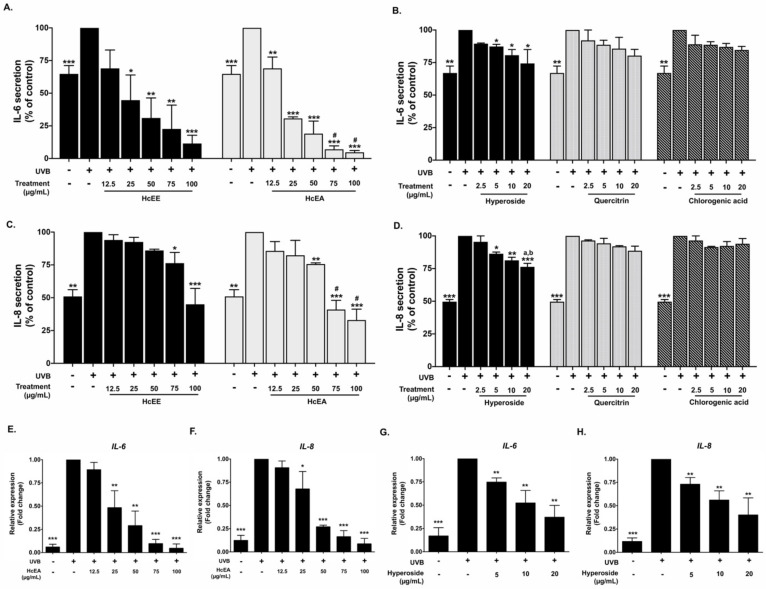
Effects of HcEE, HcEA and the main active compounds on pro-inflammatory cytokine expressions in UVB-irradiated fibroblasts. UVB-irradiated fibroblasts were treated with the extracts or active compounds for 48 h. The IL-6 and IL-8 secretions (**A**–**D**) in the culture supernatant were examined by ELISA. Cells were collected after 24 h of treatment and *IL-6* and *IL-8* mRNA levels (**E**–**H**) were determined using RT-qPCR. Data are presented as mean ± S.D. values of three independent experiments, * *p* < 0.05, ** *p* < 0.01 and *** *p* < 0.001 vs. the control UVB-irradiated fibroblasts. # *p* < 0.05 vs. HcEE at the same concentration, ^a^ *p* < 0.05 vs. quercitrin at the same concentration. ^b^ *p* < 0.05 vs. chlorogenic acid at the same concentration. HcEE: *H. cordata* ethanolic extract, HcEA: *H. cordata* ethyl acetate fraction.

Pro-inflammatory cytokines, including IL-6 and IL-8, play a key role in photo-aging and the inflammatory cascade in UV exposed skin [29]. Therefore, the effects of HcEA and hyperoside on pro-inflammatory cytokine secretions from UVB-irradiated fibroblasts were determined. UVB (15 mJ/cm^2^) irradiation increased IL-6 and IL-8 secretions by approximately 1.7-fold and 1.8-fold, respectively, when compared with non-UVB irradiated cells. The HcEE and HcEA treatments significantly inhibited UVB-induced IL-6 and IL-8 secretions in fibroblasts in a dose-dependent manner (Figure 4A,C). HcEE and HcEA at the concentration of 100 μg/mL decreased the IL-6 secretions from UVB-irradiated fibroblasts by 87% (*p* < 0.001) and 95% (*p* < 0.001), respectively when compared with the UVB-irradiated control group. Similarly, the HcEE and HcEA treatment at concentration of 100 μg/mL significantly inhibited IL-8 secretions by approximately 55% (*p* < 0.001) and 64% (*p* < 0.001), respectively. Hyperoside treatment also inhibited UVB-induced IL-6 and IL-8 secretions in fibroblasts in a dose-dependent manner (Figure 4B,D). At a concentration of 20 μg/mL, hyperoside significantly decreased IL-6 and IL-8 secretions by 25% (*p* < 0.05) and 24% (*p* < 0.001), respectively, when compared with the UVB-irradiated control group. Meanwhile, the treatments of quercitrin and chlorogenic acid at the concentrations of 20 ug/mL resulted in slightly decreased IL-6 and Il-8 secretions from UVB-irradiated fibroblasts, but statistically significant differences were not observed (*p* > 0.05).

Additionally, we confirmed the anti-inflammation properties of HcEA and hyperoside at the gene level using RT-qPCR. The results showed that UVB irradiation increased IL-6 and IL-8 mRNA levels by 5.8-fold (*p* < 0.001) and 8.3-fold (*p* < 0.001), respectively, when compared with the non-irradiated cells. The HcEA treatment significantly decreased IL-6 and IL-8 mRNA levels in a dose-dependent manner (*p* < 0.001) (Figure 4E,F). The hyperoside at the concentration of 20 ug/mL significantly decreased IL-6 and IL-8 mRNA levels by 2.7-fold (*p* < 0.01) and 2.5-fold (*p* < 0.01), respectively, when compared with the UVB-irradiated fibroblasts (Figure 4G,H). These results indicate that HcEA exhibited superior anti-inflammation properties to HcEE in UVB-irradiated fibroblasts. Moreover, hyperoside was the active compound with the strongest anti-inflammation properties among the other compounds, as determined by the ability to attenuate the pro-inflammatory cytokine secretions on UVB-irradiated dermal fibroblasts at both the gene and protein levels. Therefore, the hyperoside contents of the HcEE (33.78 ± 0.78 mg/g extract) and HcEA (170.04 ± 3.10 mg/g extract) were analyte quantified from the HPLC chromatogram (Figure 1). The quantity of hyperoside in HcEA was approximately 5 times more than the HcEE. Next, we selected the hyperoside-enriched fraction of *H. cordata* (HcEA) for other experiments.

### 2.6. HcEA and Hyperoside Increased Collagen Synthesis and Decreased MMP-1 Expression in UVB-Irradiated Dermal Fibroblasts

**Figure 5:**
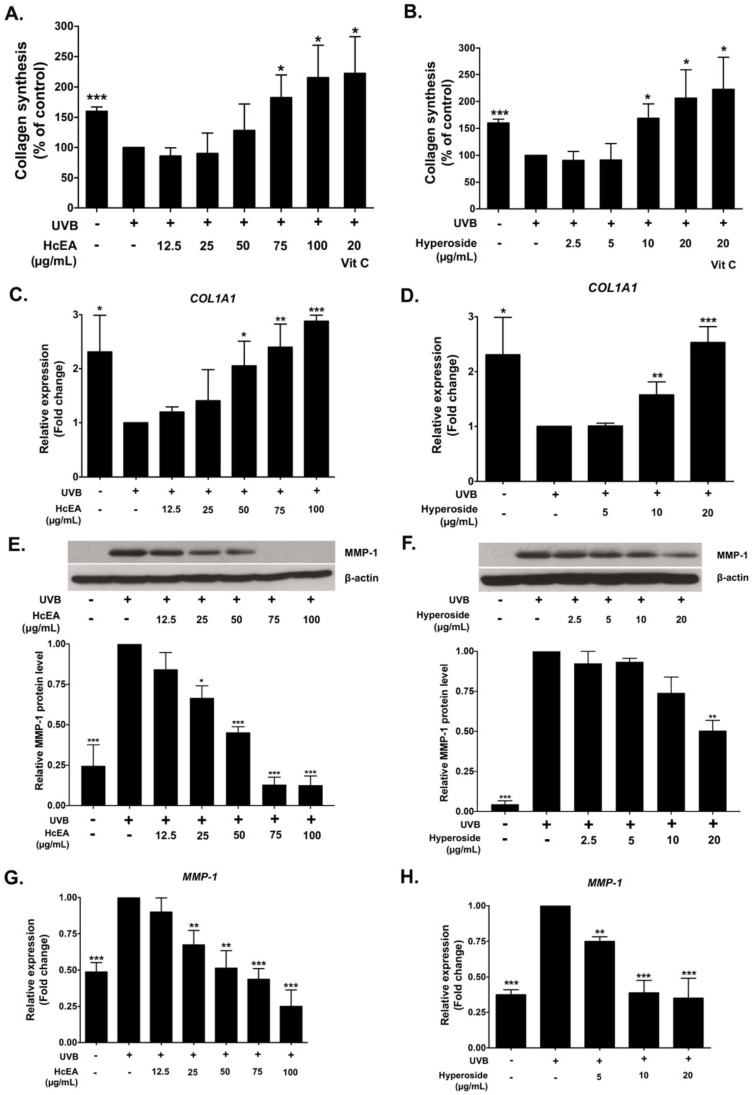
Effects of HcEA and hyperoside on collagen synthesis and MMP-1 expressions in UVB-irradiated fibroblast cells. UVB-irradiated fibroblasts were treated with HcEA or hyperoside and incubated for 48 h. The culture supernatant was collected and determined for collagen levels using a Sirius Red collagen staining kit (**A**,**B**). Cells were collected after 24 h of treatment and determined for MMP-1 expression as examined using western blotting (**E**,**F**). *COL1A1* (**C**,**D**) and *MMP-1* (**G**,**H**) mRNA levels were determined by RT-qPCR. Data are presented as mean ± S.D. values of three independent experiments, * *p* < 0.05, ** *p* < 0.01 and *** *p* < 0.001 vs. the control UVB-irradiated fibroblasts. HcEA: *H. cordata* ethyl acetate fraction, Vit C: vitamin C, MMP-1: matrix metalloproteinase-1, *COL1A1*: collagen type I gene.

Collagen plays an important role in the skin rejuvenation process and in maintaining the overall smoothness of the skin. UVB exposure inhibits collagen synthesis in the skin, leading to wrinkles, which considered a skin aging characteristic [30]. Therefore, the effects of HcEA and hyperoside treatments on collagen synthesis in UVB-irradiated fibroblasts were examined using a Sirius Red collagen staining kit. The results indicate that UVB irradiation (15 mJ/cm^2^) led to a reduction in collagen synthesis by 1.6-fold when compared with non-UVB irradiated fibroblasts. The vitamin C treatment at the concentration of 20 μg/mL increased the collagen production by 2.2-fold when compared with the UVB-irradiated fibroblast control group. The HcEA treatment significantly increased collagen synthesis in UVB-irradiated fibroblasts in a dose-dependent manner (Figure 5A), as a concentration 100 μg/mL of HcEA increased total collagen synthesis by 2.2-fold when compared with the UVB-irradiated control group (*p* < 0.05). The hyperoside treatment also significantly increased collagen synthesis in UVB-irradiated fibroblasts in a dose-dependent manner as shown in Figure 5B (*p* < 0.05). With regard to inhibition at the gene level, The HcEA treatment significantly increased *COL1A* mRNA levels in a dose-dependent manner as shown in Figure 5C (*p* < 0.001), as the concentration of 20 μg/mL of hyperoside increased collagen production 2.0-fold when compared with the control group (*p* < 0.05). Moreover, hyperoside treatment also significantly increased *COL1A* mRNA levels in UVB-irradiated fibroblasts (*p* < 0.001) as shown in Figure 5D.

The effects of HcEA and hyperoside on the expression of the collagen degradation enzyme MMP-1 in UVB-irradiated fibroblasts were also examined. As determined by western blot analysis, the UVB irradiation significantly increased MMP-1 protein expression in human skin fibroblasts when compared to the non-UVB irradiated fibroblasts as shown in Figure 5E,F (*p* < 0.001). As expected, the HcEA or hyperoside treatment significantly inhibited MMP-1 protein expression in UVB-irradiated fibroblasts (Figure 5E,F). Similarly, as determined by RT-qPCR, HcEA and hyperoside significantly decreased MMP-1 mRNA levels in UVB-irradiated fibroblast (*p* < 0.001) as shown in Figure 5G,H. Taken together, these data indicate that hyperoside in HcEA might play an important role in the induction of collagen synthesis and the inhibition of MMP-1 expression in UVB-irradiated fibroblasts at both the gene and protein levels. The potential anti-skin aging mechanisms of the hyperoside-containing *H. cordata* extract should be considered.

### 2.7. HcEA Exhibited Photoprotective Skin-Aging Properties through the Inhibition of MAPK Signaling Pathway in UVB-Irradiated Dermal Fibroblasts

**Figure 6:**
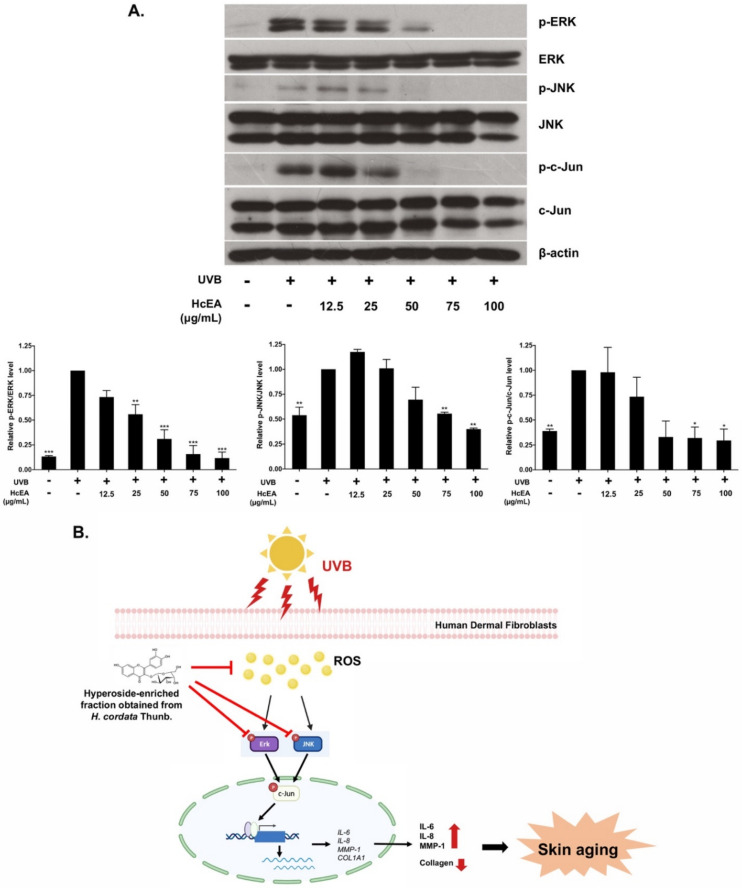
The HcEA fraction inhibited MAPK signaling pathway and AP-1 activation in UVB-irradiated fibroblast cells. UVB-irradiated fibroblasts were treated with HcEA for 24 h. Cells were kept and determined for the phosphorylation of the MAPK proteins (ERK and JNK) and activation of AP-1 transcription factor (c-JUN) using western blot analysis (**A**). The mechanism of HcEA inhibited skin-aging in UVB-irradiated human dermal fibroblasts (**B**). Data are presented as mean ± S.D. values of three independent experiments, * *p* < 0.05, ** *p* < 0.01 and *** *p* < 0.001 vs. the control group of UVB-irradiated fibroblasts. HcEA: *H. cordata* ethyl acetate fraction, UVB: ultraviolet-B, ROS: reactive oxygen species, Erk: extracellular signal-regulated kinase, JNK: c-Jun N-terminal kinase: MMP-1: matrix metalloproteinase-1, COL1A1: collagen type I gene.

Activation of the MAPK signaling pathway is partly responsible for UVB-induced photo aging in the human skin. Therefore, the inhibition of machinery proteins that contributing to MAPK pathway activation might be a useful strategy for preventing photoaging [29,31]. As shown in Figure 6A, UVB irradiation promoted the phosphorylation of the ERK and JNK proteins in dermal fibroblasts when compared with the non-UVB irradiated group. The HcEA fraction treatment dramatically inhibited the activation of ERK and JNK in UVB-irradiated fibroblast in a dose-dependent manner. Moreover, the HcEA fraction significantly inhibited the phosphorylation of the c-Jun protein which could indicate inhibition of the AP-1 transcription factor activation. Therefore, the results suggested that the HcEA fraction may protect UVB-induced photoaging through the inhibition of the MAPK signaling pathway, resulting in suppression of AP-1 activity through the inhibition of phosphorylation of c-Jun protein in UVB-treated human dermal fibroblasts (Figure 6B).

## 3. Discussion

UV radiation contributes up to 80% of factor causing prematurely aged skin [17]. Skin aging is characterized by dry skin, dullness, lack of elasticity, and fine wrinkles. Histological features include epidermal atrophy, reductions in the number of dermal fibroblasts and collagen fibers, slackening, thinness, and even function disorganization [32,33]. As UVC cannot reach the surface of the Earth, UVB and UVA are the key components of UV-associated skin damage, leading to numerous pathological manifestations of the skin, and inflammation is a crucial process among these UV-induced effects [1].

Polyphenols-containing plant extracts hold promise as they have previously exhibited a range of beneficial effects on skin in in vitro and in vivo studies. Briefly, the ethanolic extract of *Spirulina platensis* inhibited UVB irradiation-induced intracellular-ROS in human dermal fibroblasts [34]. The treatment of dermal fibroblasts with *Epilobium angustifolium* polyphenol extract resulted in a downregulation of the UV-induced release of MMP-1 and MMP-3 [35]. Hawthorn (*Crataegus spp. L.* or Crataegus pinnatifida Bge. var. major N. E. Br.) polyphenol extract, at dosages of 100 or 300 mg/kg body weight, suppressed MMP expression and stimulated the production of type I procollagen in the dorsal skin of UVB-irradiated mice [7].

Previous studies have reported that the leaves part of *H. cordata* contained more flavonoids than the roots and all other parts of the plant [19,22]. Therefore, to obtain the greatest quantity of phytochemicals from *H. cordata*, we considered choosing the leaves part of *H. cordata* to establish the ethanolic extract. The process of solvent-partition was used to establish the concentrated-extract called HcEA. Previous studies also supported the use of the ethyl acetate fraction of *H. cordata*, as the functional compounds identified by UPLC-PDA and LC/MS analysis were increased in the ethyl acetate fraction when compared with its ethanolic extract [20,36]. As determined by HPLC technique, the phenolic and flavonoid compounds of *H. cordata* identified in our study were quercitrin (quercetin-3-rhamnoside), hyperoside (quercetin-3-D-galactoside), chlorogenic acid, and rutin (quercetin-3-rutinoside). Previous studies determined by either HPLC or the UV-visible detection technique that the active compounds in *H. cordata* include chlorogenic acid, rutin, hyperoside, and quercitrin, which coincided with the finding of our study [20,24,25].

With regard to the active compounds in *H. cordata* extracts, our study demonstrated that the quercitrin in *H. cordata* was found to be the highest in quantity among all other active compounds (hyperoside, chlorogenic acid, and rutin). This finding was consistent with that of another study investigating the flavonoids in *H. cordata* obtained from different geographic origins in China [24]. Nevertheless, in our study, quercetin (one of the active flavonoids that had been identified previously in *H. cordata* [24,37]) was detected in neither HcEE nor its HcEA fraction. The reason for this might be a variation in quercitrin quantity based on the morphological traits of *H. cordata* plant flavonoids. Furthermore, other studies have reported that little or no quercetin was identified in *H. cordata*. [38,39,40].

It has been suggested that flavonoids obtained from plants can potentially protect against harmful UV irradiation by interacting with ROS through their ability to scavenge free radicals and destroy chain reactions prior to the viability of cells, resulting in the attenuation of inflammatory cytokines triggered by the oxidative metabolism because of the overproduction of ROS [20,41]. Previous studies found that flavonol glycosides obtained from *C. ternatea* flower extracts exhibited a protective effect against UV-induced oxidative stress on keratinocytes [42]. The flavonoid hesperidin exhibited strong anti-photoaging activity by regulating MMP-9 expression through the suppression of the MAPK-dependent signaling pathways in a UVB-treated hairless mice model [43]. Likewise, *H. cordata* extracts and their bioactive flavonoid molecules were previously shown to have both anti-inflammatory and antioxidant properties in both in vitro and in vivo studies. Briefly, the ethanolic extracts of *H. cordata* were found to inhibit the NF-κB signaling pathway by downregulating the phosphorylation of IκBα, and reducing TNF- α, IL-6, and IL-8 levels in phorbolmyristic acetate/calcium ionophore-induced human HMC-1 mast cells [8]. Hyperoside and quercitrin from *H. cordata* extracts attenuated LPS-induced lung inflammation in mice by oral administration [22].

The UVB-irradiation model in human dermal fibroblasts established in this study was modified from our previously described protocol [34], Specifically, this model was designed not to drastically cause cell death (with a %of cell viability of more than 80%) but would be sufficient to trigger ROS production and inflammatory responses as well as other photo-skin aging characteristics. The strong antioxidant properties of flavonoid-containing plant extracts can delay the aging process of the skin by counteracting the photo-aging process through the reduction in inflammatory responses [20,41]. Our study found that, when compared with HcEE, HcEA exhibited stronger antioxidant properties, as determined by chemical-based antioxidant assays and intracellular ROS inhibition in UVB-irradiated dermal fibroblasts. Moreover, HcEA also attenuated pro-inflammatory cytokine secretions in UVB-irradiated dermal fibroblasts, as seen by significant reductions in IL-6 and IL-8 expressions in both mRNA and protein levels.

Considering the active compounds in HcEA, to investigate the anti-skin aging properties from the active compounds of *H. cordata*, their concentration ranges (hyperoside, quercitrin, and chlorogenic acid) were calculated based on the concentrations that were determined by HPLC analysis. Therefore, in our experiments, a concentration of 0–20 μg/mL of the active compounds was representative of the amount that was identified in HcEA at a concentration of 0–200 μg/mL. Then, we further investigated whether the active compound was likely responsible for the antioxidant and anti-inflammatory properties. The results indicated that when compared among the active compounds in HcEA, hyperoside exhibited remarkable inhibitory effects on intracellular ROS inhibition and on the attenuation of cytokine gene and protein expressions. The inhibitory effects of these assays coincided with increasing concentrations of hyperoside (in a dose-dependent manner) and were significantly stronger than for the other active compounds at the same concentration.

Hyperoside is a flavonol glycoside mainly present in plants, including *Houttuynia cordata* Thunb. and plants of the genera *Hypericum* and *Crataegus* [44,45]. It exhibits several pharmacological activities, including anti-inflammatory and antioxidant effects in various experimental models. Briefly, hyperoside inhibited COX-2 and hyaluronidase enzymes and suppressed the production of IL-6, TNF-alpha, and nitric oxide in LPS-stimulated mouse peritoneal macrophages [46]. Hyperoside dose-dependently inhibited LPS-induced proliferation, migration, and inflammatory responses by suppressing the activation of the NF-κB signaling pathway, which contributes to the anti-inflammatory effect in collagen-induced arthritis [47]. It was unexpected that the second-most abundant active compound in *H. cordata*, hyperoside, exhibited stronger antioxidant and anti-inflammation capabilities in UVB-irradiated fibroblasts than quercitrin. This finding emphasized the importance of investigating the bioactivity of certain active compounds, in addition to identifying the active compounds present in the extract, since the most abundant active compounds in the extract do not necessarily possess the most potent bioactivities.

The effects of natural flavonoids on MMP-1 activity and MMP-1 expression were studied in order to establish their therapeutic potential for many inflammation-related disorders including skin photoaging [18,44,48]. Despite the importance of the MMP-1 and collagen breakdown mechanisms, only a few studies have reported on the effects of flavonoids on MMP-1. Flavonoids, such as genistein and baicalein, were reported to down-regulate MMP-1 expression in UV-irradiated human skin or UV-irradiated human dermal fibroblasts [18,49]. In our study, hyperoside exhibited the most promising inhibitory effect on skin photoaging. Therefore, we decided to further investigate the effects of HcEA and hyperoside on collagen synthesis and the proteolytic enzyme, MMP-1, in UVB-irradiated fibroblasts. The results indicates that HcEA and its active compound, hyperoside, dose-dependently decreased MMP-1 gene and protein expressions and increased the *COL1A1* expression and collagen synthesis in dermal fibroblasts upon exposure to UVB radiation.

Oxidative stress plays an important role in the signaling pathway involved in skin aging and various UVB-induced skin damaging processes. Its main feature is that it can increase intracellular ROS [20,32,41]. The accumulation of ROS can cause DNA damage, induce skin inflammatory responses, reduce antioxidant enzymes activity, influence specific survival signaling MAPK-dependent pathways, activate the AP–1 to inhibit collagen production, and increase the number of matrix metalloproteinases to decompose collagen and binding proteins in the dermis, which eventually leads to skin aging [12,14,18]. Therefore, we hypothesized that the hyperoside-enriched fraction prepared from *H. cordata* (HcEA) exhibited protective effects on UVB-induced skin aging through the modulation of the MAPK signaling pathway and the AP-1 nuclear transcription factor. Our study found that HcEA protected against collagen breakdown partly by MMP-1 inhibition, through the downregulation of MMP-1 expression in UVB-irradiated dermal fibroblasts. The present study also reported that MMP-1 downregulation and inhibition of the inflammatory response of HcEA fraction were mediated by the inhibition of MAPK signaling proteins (ERK and JNK proteins) resulting in the suppression of AP-1 activity through the inhibition of the phosphorylation of c-Jun in UVB-treated human dermal fibroblasts.

## 4. Materials and Methods

### 4.1. Chemical and Reagents

Dulbecco’s Modified Eagle Medium (DMEM) was purchased from Gibco (Grand Island, NY, USA). Fetal bovine serum (FBS) was purchased from Thermo Scientific (Waltham, MA USA). Sirius Red/Fast Green collagen staining kits were purchased from Chondrex, Inc. (Redmond, WA, USA). Sulforhodamine B reagent, 2′,7′-Dichlorofluorescin diacetate (DCF-DA), chlorogenic acid, rutin, hyperoside, quercetin, quercitrin and anti b-actin were obtained from Sigma-Aldrich (St. Louis, MO, USA). TRI reagent^®^ was purchased from Merck Millipore (Billerica, MA, USA.). ReverTra Ace^®^ qPCR Master Mix was purchased from Toyobo Co., Ltd. (Osaka, Japan). SensiFAST SYBR Lo-ROX Kit was purchased from Meridian Bioscience^®^ (Cincinnati, OH, US). The anti-MMP-1, anti-phospho-ERK, anti-ERK, anti-phospho-JNK, anti-JNK, anti-phospho-c-Jun, anti-c-Jun primary antibody and horseradish peroxidase-conjugated anti-mouse- or anti-rabbit-IgG were purchased from Cell Signaling Technology (Danvers, MA, USA).

### 4.2. Herb Materials

*H. cordata* Thunb. was harvested in 2020 from a local farm located in Chiang Mai Province, Thailand. The voucher specimen of *H. cordata* (No. 002360) was certified by the herbarium at the Flora of Thailand, Faculty of Pharmacy, Chiang Mai University.

### 4.3. Preparation of Herbal Extracts and Solvent-Partitioned Extraction Technique

**Figure 7:**
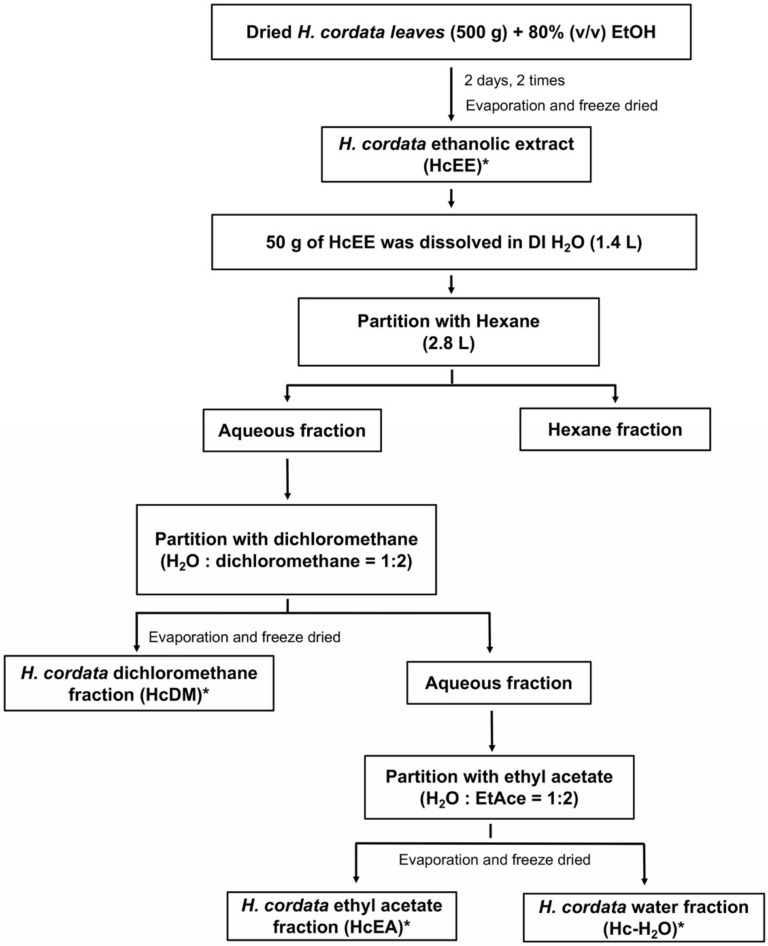
Preparation of *H. cordata* Thunb. extracts and solvent-partitioned extraction technique. The diagram represents the flow chart of ethanolic extraction and the solvent-partition method to obtain the ethanolic extract and the concentrated fractions of *H. cordata*, including, *H. cordata* ethanolic extract (HcEE), *H. cordata* dichloromethane fraction (HcDM), *H. cordata* ethyl acetate fraction (HcEA), and *H. cordata* water fraction (Hc-H_2_O). EtOH: ethanol, DI H_2_O: deionized water, EtAce: ethyl acetate. * *H. cordata* extracts that were used in this study.

Initially, 500 grams of the dried *H. cordata* leaves were soaked in 8 L of 80% (*v*/*v*) ethanol and were mixed using digital overhead stirrer (IKA^®^ RW 20, Staufen, Germany) at 500 rpm overnight at room temperature. After filtration, the extract was evaporated using a rotary vacuum evaporator at 56 °C (Buchi^®^, Flawil, Switzerland) in order to obtain ethanolic extract. The extract was resuspended in 100 mL of deionized water (DI H_2_O) and then freeze-dried to obtain the *H. cordata* ethanolic extract (HcEE). For the solvent-partitioned purification step as modified from previously described protocol [50], the ethanolic extract of *H. cordata* (50 g) was dissolved in 1.4 L of DI H_2_O and subsequently partitioned with 2.8 L of hexane. The hexane was then separated and evaporated. Afterwards, the water fraction was partitioned with a dichloromethane:water fraction, at a ratio of 2:1. Next, the dichloromethane fraction was collected, evaporated, and lyophilized, and named the *H. cordata* dichloromethane fraction (HcDM). The water fraction was then partitioned again with an ethyl acetate:water fraction, at a ratio of 2:1. The ethyl acetate fraction was then collected, evaporated, lyophilized, and named the *H. cordata* ethyl acetate fraction (HcEA). Finally, the residual fraction was collected, evaporated, and lyophilized, and named the *H. cordata* water fraction (Hc-H_2_O). A diagram of *H. cordata* extraction and solvent-partitioned extraction is shown in Figure 7. Each fraction was kept at −20 °C for further experimentation.

### 4.4. Total Phenolic Content

The total phenolic content of herbal extracts used in this study was determined by the modified Folin–Ciocalteu assay, as previously described [51]. The total phenolic content was shown as milligrams of GA equivalents per gram of extract (mg GAE/g extract).

### 4.5. Total Flavonoid Content

Total flavonoid contents were measured using the aluminum chloride (AlCl_3_) colorimetric assay, with slight modifications [52]. Each concentration of the herbal extract (250 μL) was mixed with 5% NaNO_2_ (125 μL) for 5 minutes. A total of 10% AlCl_3_ (125 μL) was then added to the mixture. Next, 1.0 mL NaOH was added, and the mixture was incubated for 15 minutes at room temperature. The absorbance of the mixture was measured at 510 nm using a spectrophotometer and then compared with the standard catechin. The total flavonoid content was expressed as mg catechin equivalents (CE) per gram of extract (mg CE/g extract).

### 4.6. Identification of Active Compounds Using HPLC

Prior to the HPLC analysis, the optimal analytical wavelength for the identification of active compounds was determined by a Shimadzu^®^ UV-1800 UV/Visible scanning spectrophotometer (Kyoto, Japan). Analyses of chlorogenic acid, rutin, hyperoside, quercitrin, and quercetin in the *H. cordata* extracts were performed using HPLC (Infinity 1260, Agilent Tecnologies, Santa Clara, CA, USA) with a reversed-phase C18 column (Zorbax Eclipse Plus C18, 250 mm × 4.6 mm, 5µm) and a pre-column (Zorbax Eclipse Plus-C18, 12.5 mm × 4.6 mm, 5µm). The HPLC condition was modified from the previously described protocol [53]. In brief, 0.1% CH_3_COOH (mobile phase A) and MeCN (mobile phase B) were the components of the mobile phase. The pre-injection time was set as 95%A:5%B. The gradient elution was set as follows: 0-8 min, from 95 to 75% of A; 8.01-32 min from 75 to 65% of A; 32.01–40 min from 65 to 5% of A; 40.01–42 min stable at 5% of A, and 42.01–45 min from 5 to 95% of A. The detection wavelength was 330 nm. The flow rate was set to 0.8 mL/min for 45 minutes. The injection volume was 10 μL. The peak area was calculated and compared with the standard to establish the concentration for each detected compound (mg/g extract).

### 4.7. ABTS and DPPH Assays for Antioxidant Properties

The antioxidant activity of the *H. cordata* extracts were determined using ABTS assay, as has been previously described [54,55]. Absorbance was recorded at 734 nm using a spectrophotometer and compared to a calibration curve of the vitamin E standard which was the positive control for the experiment.

The DPPH radical scavenging assay was determined as has been previously described [54,55]. Briefly, the 20 μL of various concentrations of *H. cordata* extracts were mixed with 180 μL of freshly prepared DPPH methanolic solution and kept in the dark for 30 minutes. Then, absorbance was measured at 540 nm. Vitamin E was used as a positive control.

### 4.8. Primary Cell Cultures

Primary human skin fibroblasts were aseptically isolated from an abdominal scar after a surgical procedure involving a cesarean delivery at the surgical operation room of Chiang Mai Maharaj Hospital, Chiang Mai University, Chiang Mai, Thailand (Study code: BIO-2558-03549 approved by Medical Research Ethics Committee, Chiang Mai University). The fibroblast cells were isolated as has been previously described [34]. The fibroblasts were cultured in DMEM supplemented with 10% FBS, 2 mM L-glutamine, 50 U/mL penicillin, and 50 μg/mL streptomycin. Cells were maintained in a 5% CO_2_ humidified incubator at 37 °C.

### 4.9. Cell Viability Assay

The cytotoxicity of the *H. cordata* extract and the active compounds against skin fibroblast cells was measured using SRB assay, as has been previously described [34]. Briefly, fibroblast cells (8 × 10^3^ cells/well) were seeded in a 96-well plate and incubated at 37 °C in 5%CO_2_ overnight. After that, the cells were treated with or without various concentrations of *H. cordata* extract (0–200 μg/mL) or active compounds (0–50 μg/mL) for 48 h. For the UVB induction model, cells were pre-treated with various concentrations of *H. cordata* extract or the active compounds for 4 h and exposed to UVB radiation at 15 mJ/cm^2^ using a CL-1000 ultraviolet crosslinker (Analytik Jena, Upland, CA, USA). The cells were then incubated with or without *H. cordata* extract or active compounds for a further 48 h. The absorbance for the SRB assay was measured at 510 nm using a microplate reader. Cell viability was calculated compared to the control and interpreted as the % of control.

### 4.10. Intracellular ROS Determination

Intracellular ROS after UVB irradiation was determined using a DCF-DA assay, as has been previously described [34]. Fibroblasts (8.0 × 10^5^ cells/well) were seeded in a 96-well plate for 24 h. Next, the cells were pre-treated with various concentrations of *H. cordata* extract, the active compounds, or vitamin C (20 μg/mL as the positive control) for 4 h and exposed to UVB radiation. The cells were then treated with or without increasing concentrations of *H. cordata* extract, the active compounds, or vitamin C for 24 h. Subsequently, the cells were washed with PBS and incubated with 10 µM of DCF-DA for a further 30 minutes. The fluorescent intensity was measured at an excitation wavelength of 485 nm and an emission wavelength of 525 nm. The intracellular ROS was calculated compared to the control group of UVB-irradiated fibroblasts.

### 4.11. Determination of Cytokine Secretion

Secretions of IL-6 and IL-8 in the cultured medium were determined using an ELISA kit (Biolegend, San Diego, CA, USA) following the manufacturer’s instructions. Fibroblasts were seeded in a 6-well-plate for 24 h. After that, the cells were pre-treated with various concentrations of *H. cordata* extract or active compounds for 4 h and then exposed to UVB for 48 h. The cultured medium was collected for ELISA testing, and the absorbance was measured at 450 and 570 nm. The cytokine secretions in the cultured medium were calculated and compared for each standard curve (IL-6 and IL-8).

### 4.12. Collagen Synthesis Assay

Primary fibroblasts were seeded into 24-well plates for 24 h and incubated at 37 °C in an atmosphere of 5% CO_2_. After incubation, the cells were pre-treated with 0.5% FBS DMEM medium for 24 h. The medium was then removed and pre-treated with various concentrations of HcEA, hyperoside, or vitamin C (positive control) for 4 h and then exposed to UVB. After 48 h of incubation, the cultured medium was kept and the collagen content was determined using a Sirius Red collagen staining kit (Chondrex Inc., Redmond, WA, USA). The protocol was followed according to the manufacturer’s instructions, which were previously described elsewhere [24]. The collagen pellets were dissolved in an extraction buffer and were measured at 540 nm using a microplate reader. The collagen in each sample was calculated according to the collagen standard curve and interpreted as the % of control.

### 4.13. Expression of IL-6, IL-8, COL1A1 and MMP1 Genes by RT- qPCR Analysis

In order to determine inflammatory cytokine (*IL-6* and *IL-8*), *MMP-1* and *COL1A1* gene expressions, fibroblasts were pre-treated with various concentrations of HcEA or hyperoside for 4 h and then exposed to UVB. After 24 h of incubation, total mRNA was isolated using TRI reagent^®^. The concentration and purity of total RNA were detected using NanoDrop™ 2000/2000c spectrophotometers (Thermo Fisher Scientific, Waltham, MA, USA) (A260/A280 > 1.8 indicating pure RNA). The cDNA was obtained via reverse transcription using a Mastercycler^®^ nexus gradient machine (Eppendorf, Hamburg, Germany). The quantitative real-time PCR technique was determined using a qRT-PCR ABITM 7500 Fast and 7500 Real-Time PCR machine (Thermo Fisher Scientific, Waltham, MA, USA). Gene expressions were analyzed using QuantStudio 6 Flex real-time PCR system software (Applied Biosystems, Waltham, MA, USA). The 2^−ΔΔCT^ method with normalization to GAPDH and controls were used for the calculation of results. All primer sequences used in this study were as follows: *IL-6* forward: 5′-ATG AAC TCC TTC ACA AGC-3′, reverse: 5′-GTT TTC TGC CAG TGC CTC TTT G-3′ (Bio Basic Canada Inc., Ontario, Canada). *IL-8* forward, 5′-CTC TGT GTG AAG GTG CAG TTT TG-3′, reverse, 5′-TCT CTT CCA TCA GAA AGC TTT ACA ATA-3′; *COL1A1* forward, 5′-TGA GCC AGC AGA TCG AGA-3′ reverse, 5′-ACC AGT CTC CAT GTT GCA GA-3′; *MMP1* forward, 5′-TCT GAC GTT GAT CCC AGA GAG CAG-3 reverse, 5′-CAG GGT GAC ACC AGT GAC TGC AC-3′and *GAPDH* forward, 5′-TCA ACA GCG ACA CCC AC-3′ reverse, 5′-TCA ACA GCG ACA CCC AC-3′ (Humanizing Genomics Macrogen, Geumcheon-gu, Seoul, South Korea) [56,57,58].

### 4.14. Western Blot Analysis

In order to determine the effects of HcEA on MMP-1 expression and MAPK signaling in UVB-irradiated fibroblasts, fibroblasts were pre-treated with various concentrations of HcEA for 4 h and then exposed to UVB (15 mJ/cm^2^). After 24 h of incubation, cells were collected and lysed using RIPA buffer. the protein concentration was determined using the Bradford method. The whole-cell lysate was subjected to 12% SDS-PAGE. The separated proteins were transferred into nitrocellulose membranes. The membranes were blocked with 5% non-fat dried milk protein in 0.5% TBS-tween. After that, the membranes were washed twice with 0.5% TBS-tween. Then, the membranes were further incubated overnight with the primary antibody at 4 °C. Next, the membranes were washed 5 times with 0.5% TBS-tween followed by incubating with horseradish peroxidase-conjugated anti-mouse or rabbit-IgG, depending on the primary antibody, at room temperature for 2 h and were then washed 5 times with 0.5% TBS-tween. The bound antibodies were detected using the chemiluminescent detection system and then exposed to X-ray film (GE Healthcare Ltd., Little Chalfont, U.K.). Equal values of protein loading were confirmed as each membrane was stripped and re-probed with anti-β-actin antibody. Band density levels were analyzed using IMAGE J 1.410.

### 4.15. Statistical Analysis

All data are presented as mean ± standard deviation (S.D.) values. Statistical analysis was performed with Prism version 8.0 software, using an independent t-test and a one-way ANOVA with Dunnett’s test. Statistical significance was determined at *p < 0.05, **p < 0.01 and ***p < 0.001 vs. the control of each experiment. #*p* < 0.05 vs. HcEE at the same concentration. ^a^*p* < 0.05 vs. quercitrin at the same concentration. ^b^*p* < 0.05 vs. chlorogenic acid at the same concentration.

## 5. Conclusions

This is the first report to demonstrate that the hyperoside-enriched fraction prepared from an extract from the leaves of *H. cordata* potentially exhibited a protective effect on UVB-induced skin photoaging in human dermal fibroblasts through their antioxidant and anti-inflammation capabilities. Furthermore, HcEA can induce collagen synthesis and inhibit MMP-1 expression via the modulation of the MAPK pathway and the AP-1 nuclear transcription factor. The results obtained from this study provide evidence that can be applied in the cosmetic industry, as *H. cordata* extract can potentially be used in pharmaceutical or cosmetic formulations as a photoprotective or anti-aging skin agent.

## Figures and Tables

**Table 1:** Total phenolic contents, total flavonoid contents, and antioxidant activities of *H. cordata* extracts.

*H. cordata* Extracts	Polyphenols		Antioxidant Capacity	
	Total Phenolic Content (mg GAE/g Extract)	Total Flavonoid Content (mg CE/g Extract)	IC_50_ DPPH Assay (μg/mL)	IC_50_ ABTS Assay (μg/mL)
HcEE	172.57 ± 6.73	126.97 ± 7.44	41.95 ± 2.29	17.36 ± 0.84
HcDM	113.08 ± 9.24	97.23 ± 10.16	113.94 ± 10.70	26.69 ± 1.40
HcEA	591.01 ± 7.11 **	433.86 ± 10.16 **	17.64 ± 2.25 **	4.96 ± 0.24 **
Hc-H_2_O	128.07 ± 4.40	90.09 ± 6.78	66.35 ± 3.84	19.72 ± 1.75
Vitamin E			33.97 ± 1.62	14.95 ± 0.41

Data are presented as mean ± S.D. values of three independent experiments. ** *p* < 0.01 vs. other *H. cordata* Thunb. extracts (HcEE, HcDM, and Hc-water). GAE: gallic acid equivalents, CE: catechin equivalents, HcEE: *H. cordata* ethanolic extract, HcDM: *H. cordata* dichloromethane fraction, HcEA: *H. cordata* ethyl acetate fraction, Hc-H_2_O: *H. cordata* water fraction.

## Data Availability

Data sharing is not applicable to this article.

## References

[1] Rabe J.H., Mamelak A.J., McElgunn P.J., Morison W.L., Sauder D.N. (2006). Photoaging: Mechanisms and repair. J. Am. Acad. Dermatol..

[2] Rodier F., Campisi J. (2011). Four faces of cellular senescence. J. Cell Biol..

[3] Cadet J., Wagner J.R. (2013). DNA base damage by reactive oxygen species, oxidizing agents, and UV radiation. Cold Spring Harb. Perspect. Biol..

[4] Kageyama H., Waditee-Sirisattha R. (2019). Antioxidative, Anti-Inflammatory, and Anti-Aging Properties of Mycosporine-Like Amino Acids: Molecular and Cellular Mechanisms in the Protection of Skin-Aging. Mar. Drugs.

[5] Gegotek A., Domingues P., Skrzydlewska E. (2018). Proteins involved in the antioxidant and inflammatory response in rutin-treated human skin fibroblasts exposed to UVA or UVB irradiation. J. Dermatol. Sci..

[6] Yaar M., Gilchrest B.A. (2007). Photoageing: Mechanism, prevention and therapy. Br. J. Dermatol..

[7] Liu S., You L., Zhao Y., Chang X. (2018). Hawthorn Polyphenol Extract Inhibits UVB-Induced Skin Photoaging by Regulating MMP Expression and Type I Procollagen Production in Mice. J. Agric. Food Chem..

[8] Choi S., Youn J., Kim K., Joo da H., Shin S., Lee J., Lee H.K., An I.S., Kwon S., Youn H.J. (2016). Apigenin inhibits UVA-induced cytotoxicity in vitro and prevents signs of skin aging in vivo. Int. J. Mol. Med..

[9] Shin S., Cho S.H., Park D., Jung E. (2020). Anti-skin aging properties of protocatechuic acid in vitro and in vivo. J. Cosmet. Dermatol..

[10] Aggarwal B.B., Shishodia S., Sandur S.K., Pandey M.K., Sethi G. (2006). Inflammation and cancer: How hot is the link?. Biochem. Pharmacol..

[11] Rastogi R.P., Richa, Kumar A., Tyagi M.B., Sinha R.P. (2010). Molecular mechanisms of ultraviolet radiation-induced DNA damage and repair. J. Nucleic Acids.

[12] Domaszewska-Szostek A., Puzianowska-Kuznicka M., Kurylowicz A. (2021). Flavonoids in Skin Senescence Prevention and Treatment. Int. J. Mol. Sci..

[13] Lee H.J., Seo H.S., Kim G.J., Jeon C.Y., Park J.H., Jang B.H., Park S.J., Shin Y.C., Ko S.G. (2013). *Houttuynia cordata* Thunb inhibits the production of pro-inflammatory cytokines through inhibition of the NFkappaB signaling pathway in HMC-1 human mast cells. Mol. Med. Rep..

[14] Cooper S.J., Bowden G.T. (2007). Ultraviolet B regulation of transcription factor families: Roles of nuclear factor-kappa B (NF-kappaB) and activator protein-1 (AP-1) in UVB-induced skin carcinogenesis. Curr. Cancer Drug Targets.

[15] Terlecki-Zaniewicz L., Pils V., Bobbili M.R., Lammermann I., Perrotta I., Grillenberger T., Schwestka J., Weiss K., Pum D., Arcalis E. (2019). Extracellular Vesicles in Human Skin: Cross-Talk from Senescent Fibroblasts to Keratinocytes by miRNAs. J. Invest. Dermatol..

[16] Battie C., Jitsukawa S., Bernerd F., Del Bino S., Marionnet C., Verschoore M. (2014). New insights in photoaging, UVA induced damage and skin types. Exp. Dermatol..

[17] Amaro-Ortiz A., Yan B., D’Orazio J.A. (2014). Ultraviolet radiation, aging and the skin: Prevention of damage by topical cAMP manipulation. Molecules.

[18] Lim H., Kim H.P. (2007). Inhibition of mammalian collagenase, matrix metalloproteinase-1, by naturally-occurring flavonoids. Planta Med..

[19] Kumar M., Prasad S.K., Hemalatha S. (2014). A current update on the phytopharmacological aspects of *Houttuynia cordata* Thunb. Pharm. Rev..

[20] Shingnaisui K., Dey T., Manna P., Kalita J. (2018). Therapeutic potentials of *Houttuynia cordata* Thunb. against inflammation and oxidative stress: A review. J. Ethnopharmacol..

[21] Park E., Kum S., Wang C., Park S.Y., Kim B.S., Schuller-Levis G. (2005). Anti-inflammatory activity of herbal medicines: Inhibition of nitric oxide production and tumor necrosis factor-alpha secretion in an activated macrophage-like cell line. Am. J. Chin. Med..

[22] Lee J.H., Ahn J., Kim J.W., Lee S.G., Kim H.P. (2015). Flavonoids from the aerial parts of *Houttuynia cordata* attenuate lung inflammation in mice. Arch. Pharm. Res..

[23] Yun M.E., Lee Y.S., Lee Y.J., Park Y.M., Park S.N. (2018). Antimicrobial, antioxidant and cellular protective effects of *Houttuynia cordata* extract and fraction. Appl. Chem. Eng..

[24] Ling-Shang W.U., Jin-Ping S.I., Xiao-Qing Y.U.A.N., Xue-Rong S.H.I. (2009). Quantitive variation of flavonoids in *Houttuynia cordata* from different geographic origins in China. Chin. J. Nat. Med..

[25] Nguyen V., Le V., Vo T., Bui L., Anh H., Danh V. (2020). Preliminary Phytochemical Screening and Determination of Total Polyphenols and Flavonoids Content in the Leaves of Houttuynia cordata Thunb, Proceedings of the IOP Conference Series: Materials Science and Engineering, Penang, Malaysia, 17–19 July 2019.

[26] Tian L., Shi X., Yu L., Zhu J., Ma R., Yang X. (2012). Chemical composition and hepatoprotective effects of polyphenol-rich extract from *Houttuynia cordata* tea. J. Agric. Food Chem..

[27] Ling L.J., Lu Y., Zhang Y.Y., Zhu H.Y., Tu P., Li H., Chen D.F. (2020). Flavonoids from *Houttuynia cordata* attenuate H1N1-induced acute lung injury in mice via inhibition of influenza virus and Toll-like receptor signalling. Phytomedicine.

[28] Lee H., Sung J., Kim Y., Jeong H.S., Lee J. (2019). Protective effects of unsaponifiable matter from perilla seed meal on UVB-induced damages and the underlying mechanisms in human skin fibroblasts. Antioxidants.

[29] Subedi L., Lee T.H., Wahedi H.M., Baek S.H., Kim S.Y. (2017). Resveratrol-Enriched Rice Attenuates UVB-ROS-Induced Skin Aging via Downregulation of Inflammatory Cascades. Oxid. Med. Cell Longev..

[30] Cho Y.H., Bahuguna A., Kim H.H., Kim D.I., Kim H.J., Yu J.M., Jung H.G., Jang J.Y., Kwak J.H., Park G.H. (2017). Potential effect of compounds isolated from *Coffea arabica* against UV-B induced skin damage by protecting fibroblast cells. J. Photochem. Photobiol. B.

[31] Wang L., Oh J.Y., Lee W., Jeon Y.J. (2021). Fucoidan isolated from *Hizikia fusiforme* suppresses ultraviolet B-induced photodamage by down-regulating the expressions of matrix metalloproteinases and pro-inflammatory cytokines via inhibiting NF-kappaB, AP-1, and MAPK signaling pathways. Int. J. Biol. Macromol..

[32] Cao C., Xiao Z., Wu Y., Ge C. (2020). Diet and Skin Aging-From the Perspective of Food Nutrition. Nutrients.

[33] Farage M.A., Miller K.W., Elsner P., Maibach H.I. (2013). Characteristics of the Aging Skin. Adv. Wound Care.

[34] Mapoung S., Arjsri P., Thippraphan P., Semmarath W., Yodkeeree S., Chiewchanvit S., Piyamongkol W., Limtrakul P. (2020). Photochemoprotective effects of *Spirulina platensis* extract against UVB irradiated human skin fibroblasts. South Afr. J. Bot..

[35] Ruszova E., Cheel J., Pavek S., Moravcova M., Hermannova M., Matejkova I., Spilkova J., Velebny V., Kubala L. (2013). *Epilobium angustifolium* extract demonstrates multiple effects on dermal fibroblasts in vitro and skin photo-protection in vivo. Gen. Physiol. Biophys..

[36] Chun J.M., Nho K.J., Kim H.S., Lee A.Y., Moon B.C., Kim H.K. (2014). An ethyl acetate fraction derived from *Houttuynia cordata* extract inhibits the production of inflammatory markers by suppressing NF-small ka, CyrillicB and MAPK activation in lipopolysaccharide-stimulated RAW 264.7 macrophages. BMC Compl. Altern. Med..

[37] Chiow K., Phoon M., Putti T., Tan B.K., Chow V.T. (2016). Evaluation of antiviral activities of *Houttuynia cordata* Thunb. extract, quercetin, quercetrin and cinanserin on murine coronavirus and dengue virus infection. Asian Pac. J. Trop. Med..

[38] Jang D.-S., Kim J.-M., Lee Y.-M., Yoo J.-L., Kim Y.-S., Kim J.-H., Kim J.-S. (2006). Flavonols from *Houttuynia cordata* with protein glycation and aldose reductase inhibitory activity. Nat. Prod. Sci..

[39] Fuse J.-i., Kanamori H., Sakamoto I., Yahara S. (1994). Studies on flavonol glycosides in *Houttuynia cordata*. Nat. Med..

[40] Kawamura T., Hisata Y., Okuda K., Noro Y., Tanaka T., Yoshida M., Sakai E. (1994). Pharmacognostical studies of Houttuyniae Herba (1). Flavonoid glycosides contents of *Houttuynia cordata* Thunb. Nat. Med..

[41] Shukla R., Pandey V., Vadnere G., Lodhi S. (2019). Chapter 18-Role of Flavonoids in Management of Inflammatory Disorders. Bioact. Food Diet. Interv. Arthritis Relat. Inflamm. Dis..

[42] Zakaria N., Okello E., Howes M.J., Birch-Machin M., Bowman A. (2018). In vitro protective effects of an aqueous extract of *Clitoria ternatea* L. flower against hydrogen peroxide-induced cytotoxicity and UV-induced mtDNA damage in human keratinocytes. Phytother. Res..

[43] Lee H.J., Im A.-R., Kim S.-M., Kang H.-S., Lee J.D., Chae S. (2018). The flavonoid hesperidin exerts anti-photoaging effect by downregulating matrix metalloproteinase (MMP)-9 expression via mitogen activated protein kinase (MAPK)-dependent signaling pathways. BMC Compl. Altern. Med..

[44] Kong Y., Sun W., Wu P. (2020). Hyperoside exerts potent anticancer activity in skin cancer. Front. Biosci..

[45] Zheng Y.-M., Xu X.-Y., Fu S.-Q., Yang Y.-H. (2005). Quantitative Determination of Hyperoside and Quercitrin in *Houttuynia cordata* by HPLC. Res. Pract. Chin. Med..

[46] Kim S.-J., Um J.-Y., Hong S.-H., Lee J.-Y. (2011). Anti-inflammatory activity of hyperoside through the suppression of nuclear factor-κB activation in mouse peritoneal macrophages. Am. J. Chin. Med..

[47] Jin X.N., Yan E.Z., Wang H.M., Sui H.J., Liu Z., Gao W., Jin Y. (2016). Hyperoside exerts anti-inflammatory and anti-arthritic effects in LPS-stimulated human fibroblast-like synoviocytes in vitro and in mice with collagen-induced arthritis. Acta Pharm. Sin..

[48] Mapoung S., Umsumarng S., Semmarath W., Arjsri P., Thippraphan P., Yodkeeree S., Limtrakul Dejkriengkraikul P. (2021). Skin Wound-Healing Potential of Polysaccharides from Medicinal Mushroom *Auricularia auricula-judae* (Bull.). J. Fungi.

[49] Lim H., Heo M.Y., Kim H.P. (2019). Flavonoids: Broad Spectrum Agents on Chronic Inflammation. Biomol. Ther..

[50] Arjsri P., Phetcharaburanin J., Suksawat M., Mapoung S., Subkamkaew C., Semmarath W., Yodkeeree S., Limtrakul P. (2021). *Spirogyra neglecta* (Hassall) Kützing attenuates metastasis of castration-resistant human prostate cancer via the blockage of AKT signaling pathway. South Afr. J. Bot..

[51] Sánchez-Rangel J.C., Benavides J., Heredia J.B., Cisneros-Zevallos L., Jacobo-Velázquez D.A. (2013). The Folin–Ciocalteu assay revisited: Improvement of its specificity for total phenolic content determination. Anal. Methods.

[52] Yodkeeree S., Thippraphan P., Punfa W., Srisomboon J., Limtrakul P. (2018). Skin Anti-aging Assays of Proanthocyanidin Rich Red Rice Extract, Oryzanol and Other Phenolic Compounds. Nat. Prod. Commun..

[53] Temerdashev Z., Milevskaya V., Vinitskaya E. (2021). The method of establishing the authenticity and quality of *Hypericum perforatum* L. and *Salvia officinalis* L.. MethodsX.

[54] Nenadis N., Tsimidou M. (2010). Assessing the activity of natural food antioxidants. Oxidation in Foods and Beverages and Antioxidant Applications.

[55] Lee K.J., Oh Y.C., Cho W.K., Ma J.Y. (2015). Antioxidant and anti-inflammatory activity determination of one hundred kinds of pure chemical compounds using offline and online screening HPLC assay. Evid. Based Compl. Alternat. Med..

[56] Lorrio S., Rodríguez-Luna A., Delgado-Wicke P., Mascaraque M., Gallego M., Pérez-Davó A., González S., Juarranz Á. (2020). Protective effect of the aqueous extract of *Deschampsia antarctica* (EDAFENCE^®^) on skin cells against blue light emitted from digital devices. Int. J. Mol. Sci..

[57] Yoon H., Choi S.-I., Kim E.K. (2020). Uptake of cell debris and enhanced expression of inflammatory factors in response to dead cells in corneal fibroblast cells. Exp. Eye Res..

[58] Avola R., Graziano A.C.E., Pannuzzo G., Bonina F., Cardile V. (2019). Hydroxytyrosol from olive fruits prevents blue-light-induced damage in human keratinocytes and fibroblasts. J. Cell. Physiol..

